# Disease or function? What matters most for self-rated health in older people depends on age

**DOI:** 10.1007/s40520-020-01507-1

**Published:** 2020-03-04

**Authors:** Viviane S. Straatmann, Davide L. Vetrano, Laura Fratiglioni, Amaia Calderón-Larrañaga

**Affiliations:** 1 Aging Research Center, Department of Neurobiology, Care sciences and Society (NVS), Karolinska Institutet, Stockholm University, Tomtebodavägen 18A, Floor 10, 17165 Solna, Sweden; 2 Department of Geriatrics, Catholic University of Rome, Rome, Italy; 3 Centro di Medicina dell’Invecchiamento, Fondazione Policlinico A. Gemelli, Rome, Italy; 4 Stockholm Gerontology Research Center, Stockholm, Sweden

**Keywords:** Self-rated health, Chronic diseases, Functioning, Disability, Older adults

## Abstract

**Background:**

Self-rated health (SRH) holistically captures older adults’ health status from the perspective of the individual.

**Aims:**

To explore the accuracy of five objective health indicators related to diseases, physical function, cognition and disability in discriminating SRH among the youngest and oldest old.

**Methods:**

We used baseline data from 2196 participants of the Swedish National Study on Aging and Care in Kungsholmen (SNAC-K), Sweden (years 2001–2004). Area under the receiver operating characteristic curves (AUROC) were obtained from logistic regressions adjusted by sex, age and education.

**Results:**

Among the youngest old, having ≥ 4 chronic diseases showed the highest discriminatory capacity of poor versus good SRH (AUROC: 0.714). Among the oldest old, a walking speed < 1.0 m/s showed the highest discriminatory capacity of poor versus good SRH (AUROC: 0.683), followed by ≥ 1 limitations in IADL (AUROC: 0.664).

**Conclusion:**

What matters most for SRH in older people depends on age, with walking speed playing a major role among the oldest old.

**Electronic supplementary material:**

The online version of this article (10.1007/s40520-020-01507-1) contains supplementary material, which is available to authorized users.

## Introduction

The demographic transition taking place in industrialized countries has led to an increasingly aged population, with a particular rise in the number and proportion of old people aged over 80 years [1]. As a public health response to this phenomenon, the World Health Organization (WHO) has placed emphasis on promoting healthy aging by considering the complex person-environment interactions within a more integrative understanding of health in old age, in order to optimize health outcomes in this population [2].

Self-rated health (SRH) is a multidimensional indicator that provides information about subjects’ mental and physical well-being, and it has been used to holistically assess the health status of old people from the perspective of the individual [3]. The increasing evidence that SRH is a strong predictor of both mortality and other physical and mental health outcomes [3, 4] has led researchers to investigate the determinants of SRH itself [5, 6]. Most of these studies have used self-reported measures and hardly anyone has stratified their analyses into youngest and oldest old, two groups that clearly differ in their health and social characteristics and needs [1].

In this study, we explored how different objective health indicators related to diseases, physical function, cognition and disability can discriminate among older people with varying levels of SRH, at different ages.

## Methods

We used baseline data from the Swedish National study on Aging and Care Kungsholmen (SNAC-K) including individuals 60 years or older living in the community or in institutions in central Stockholm (years 2001–2004). The sample was randomly selected from 11 age cohorts (ages 60, 66, 72, 78, 81, 84, 87, 90, 93, 96 and ≥ 99 years). Data were collected through interviews, clinical examinations and laboratory testing by trained physicians, nurses, and psychologists. The SNAC-K study was approved by the Regional Ethical Review Board in Stockholm. Written informed consent was obtained from participants or their next of kin.

SRH was assessed with the question “in general, how would you say your health is?” and further operationalized by categorizing the answers “poor” and “fair” versus “good”, “very good” and “excellent”. Out of the 3363 SNAC-K participants, we excluded those with dementia or a Mini-Mental State Examination (MMSE) score < 24 at baseline leading to a sample of 2977 participants. Data on SRH was available for 2318 (77.9%), and 2196 (73.8%) participants had complete information in all investigated variables.

We considered five objective health measures covering the health spectrum in the older population [7]: (1) cognition assessed using the MMSE, with scores ranging between 0–30 (lower scores indicate higher impairment); (2) physical function measured through walking speed when participants were asked to walk 6 m or 2.4 m if the participant reported walking quite slowly; (3) count of diseases assessed through a physician-diagnosed comprehensive list of chronic conditions; (4) severe disability measured as the number of basic activities of daily living (ADL) (bathing, dressing, toileting, etc.) a person was unable to perform independently; and (5) mild disability measured as the number of instrumental activities of daily living (IADL) (grocery shopping, meal preparation, housekeeping, etc.) a person was unable to perform independently.

Area under the receiver operating characteristic curves (AUROC) derived from logistic regressions adjusted by sex, age and education were used to estimate health indicators’ capacity to discriminate between people with poor versus good SRH. First, we identified the best cut-offs for each of the five objective health indicators in discriminating SRH. Second, we compared the discriminatory capacity of the best cut-offs for the five health indicators within the youngest (< 78 years) and oldest (≥ 78 years) subsamples. The cut-off of 78 years was used given that it divided the study sample into two equally-sized groups, while respecting the age-cohort structure of SNAC-K. The discriminatory capacity of the health indicators was finally compared considering them as continuous variables. All analyses were performed in Stata version 15 (StataCorp LP, College Station, TX, USA).

## Results

The study population was aged 60–99 years (mean 71.8 years), 61.8% were women, 37.8% had a university degree and 1.6% were living in institutions.

The best cut-offs in discriminating poor versus good SRH were ≥ 4 chronic diseases (AUROC 95% CI 0.728, 0.705–0.752), MMSE score < 29 (AUROC 95% CI 0.665, 0.640–0.691), walking speed < 1.0 m/s (AUROC 95% CI 0.729, 0.704–0.753), ≥ 1 ADL limitations (AUROC 95% CI 0.665, 0.641–0.691), and ≥ 1 IADL limitations (AUROC 95% CI 0.691, 0.666–0.716). Results for the different cut-offs tested for each of the five health indicators are presented in Supplementary Table 1.

Almost half of the participants (46% in the total study sample, 38.8% in the youngest old and 72.7% in the oldest old) had ≥ 4 chronic diseases and 27.6% (17.1% in the youngest old and 66.1% in the oldest old) were unable to walk faster than 1.0 m/s. More than half of the participants (57.8% in the total study sample, 52.2% in the youngest old and 78.4% in the oldest old) had a MMSE score < 29, and 1.9% (0.9% in the youngest old and 5.3% in the oldest old) and 9.9% (5.5% in the youngest old and 26.3% in the oldest old) were unable to perform at least one ADL or IADL independently, respectively. Among the youngest group, 23.7% rated their health as poor, and 44.7% did so in the oldest group.

**Fig. 1:**
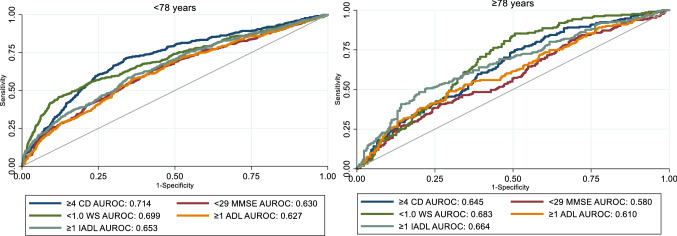
Discriminatory capacity of the five objective health indicators concerning poor versus good SRH, stratified by age group. *AUROC* area under the receiver operating characteristic curve, *CD* chronic diseases, *WS* walking speed (m/s), *MMSE* Mini-Mental State Examination, *ADL* basic activities of daily living, *IADL* instrumental activities of daily living. All estimates derived from logistic regressions adjusted by sex, age and education

For the youngest old, having ≥ 4 chronic diseases showed the highest discriminatory capacity of poor versus good SRH (AUROC 95% CI 0.714, 0.684–0.743). In the oldest group, a walking speed < 1.0 m/s showed the highest discriminatory capacity of poor SRH versus good (AUROC 95% CI 0.683, 0.635- 0.731), followed by ≥ 1 limitations in IADL (AUROC 95% CI 0.664, 0.615–0.714) (Fig. 1). All health indicators performed worse in the oldest old compared to the youngest old. Similar results were obtained when all five health indicators were operationalized as continuous variables (Supplementary Table 2).

## Discussion

In summary, different health indicators discriminate SRH differently depending on older people’s age, with the burden of chronic diseases playing a major role among the youngest old and walking speed among the oldest old. In line with our findings, a Dutch study on older adults revealed that the association between poor SRH and chronic diseases becomes weaker with increasing age, whereas the association between poor SRH and severe disability becomes stronger over time, suggesting that people may prioritize functioning well rather than having no diseases as they age [6]. According to our findings, walking speed was an even better discriminator of poor SRH than disability among the oldest old. Previous research has also found consistent associations between walking speed and SRH in the older population [5]. The fact that, in the youngest group, the burden of diseases is the best discriminator of poor SRH corroborates the findings from studies conducted in the general adult population [8] as well as in samples of older people [9].

Interestingly, the optimal cut-offs for multimorbidity (i.e., ≥ 4 chronic diseases) and walking speed (i.e., < 1 m/s) differed from more conventional definitions adopted in clinical practice and epidemiological studies (i.e., ≥ 2 chronic diseases and < 0.8 m/s, respectively). This suggests that clinically relevant cut-offs may not fully capture individuals’ self-perception of health, and calls for future studies to adopt a more patient-centered approach.

Caution is needed in interpreting the findings of our study since the predictive capacity of the different health indicators, despite being maximized by taking the thresholds with highest AUROC values, were fair or good rather than excellent. Moreover, our choice of an age cut-off prevented us from detecting gradual shifts in those indicators that matter most. Using finer age gradations was, however, not possible due to low numbers. To reduce potential measurement error of SRH, people with dementia or MMSE < 24 were excluded from our study, which may limit the generalizability of the findings to those cognitively impaired.

## Conclusions and implications

A focus on curing diseases, as is the case for most health systems around the world today, will prevent the oldest old from receiving the care they need [2]. Efforts to engage care providers and policy makers around the principles of integrated care that prioritizes the functional capacity of older people and the complex interplay of biological and non-biological factors are therefore urgently needed [10]. From a research perspective, walking speed may be a relevant outcome to be considered in observational studies as well as randomized trials, given its meaningfulness among the oldest old.

## Electronic supplementary material

Below is the link to the electronic supplementary material.

Supplementary file1 (DOCX 23 kb)
