# Supplementary material for: Disease or function? What matters most for self-rated health in older people depends on age
Source: Aging Clin Exp Res. 2020 Mar 4;32(8):1591–4. doi: 10.1007/s40520-020-01507-1 (PMC7452924; doi:10.1007/s40520-020-01507-1)
Supplement: Supplementary file 1 — Supplementary file1 (DOCX 23 kb) [file 40520_2020_1507_MOESM1_ESM.docx]

**Online Supplementary File**

*Disease or function? What matters most for self-rated health in older people depends on age*

This data supplement contains additional results of the study.

**Supplementary Table 1. Discriminatory capacity of all tested cut-off points for the five health indicators.**

**A. Chronic diseases (number)**

|  | **AUROC (95%CI)** |
| --- | --- |
| ≥1 | 0.671 (0.646-0.696) |
| ≥2 | 0.688 (0.664-0.711) |
| ≥3 | 0.718 (0.695-0.741) |
| ≥4 | 0.728 (0.705-0.752) |
| ≥5 | 0.714 (0.690-0.738) |
| ≥6 | 0.714 (0.689-0.738) |
| ≥7 | 0.699 (0.674-0.725) |
| ≥8 | 0.687 (0.662-0.703) |
| ≥9 | 0.677 (0.652-0.703) |
| ≥10 | 0.671 (0.646-0.696) |

**B. Mini Mental State Examination (score)**

|  | **AUROC (95%CI)** |
| --- | --- |
| <24 | 0.663 (0.637-0.686) |
| <25 | 0.663 (0.637-0.688) |
| <26 | 0.663 (0.637-0.688) |
| <27 | 0.663 (0.637-0.688) |
| <28 | 0.664 (0.639-0.689) |
| <29 | 0.665 (0.640-0.691) |

**C. Walking speed (meters/second)**

|  | **AUROC (95%CI)** |
| --- | --- |
| <0.1 | 0.664 (0.638-0.689) |
| <0.2 | 0.664 (0.639-0.690) |
| <0.3 | 0.667 (0.641-0.692) |
| <0.4 | 0.672 (0.646-0.698) |
| <0.5 | 0.692 (0.666-0.717) |
| <0.6 | 0.692 (0.666-0.718) |
| <0.7 | 0.717 (0.692-0.742) |
| <0.8 | 0.718 (0.692-0.743) |
| <0.9 | 0.729 (0.704-0.753) |
| <1.0 | 0.729 (0.704-0.753) |
| <1.1 | 0.715 (0.690-0.739) |
| <1.2 | 0.715 (0.690-0.739) |
| <1.3 | 0.697 (0.673-0.721) |
| <1.4 | 0.697 (0.673-0.721) |
| <1.5 | 0.697 (0.673-0.721) |

**D. Impaired activities of daily living (number)**

|  | **AUROC (95%CI)** |
| --- | --- |
| ≥1 | 0.665 (0.639-0.690) |
| ≥2 | 0.662 (0.636-0.687) |
| ≥3 | 0.662 (0.636-0.687) |
| ≥4 | 0.662 (0.636-0.687) |
| ≥5 | 0.662 (0.636-0.687) |
| ≥6 | 0.662 (0.636-0.687) |

**E. Impaired instrumental activities of daily living (number)**

|  | **AUROC (95%CI)** |
| --- | --- |
| ≥1 | 0.692 (0.666-0.717) |
| ≥2 | 0.686 (0.660-0.711) |
| ≥3 | 0.673 (0.648-0.699) |
| ≥4 | 0.667 (0.642-0.699) |
| ≥5 | 0.665 (0.639-0.691) |
| ≥6 | 0.664 (0.638-0.689) |
| ≥7 | 0.664 (0.638-0.689) |
| ≥8 | 0.663 (0.638-0.689) |

**Supplementary Table 2. Discriminatory capacity of the five health indicators (as continuous variables) for SRH, by age group.**

|  | **<78 years** | **≥78 years** |
| --- | --- | --- |
|  | **AUROC (95%CI)** | **AUROC (95%CI)** |
| ≥4 CD | 0.745 (0.717-0.772) | 0.689 (0.641-0.736) |
| ≤29 MMSE | 0.629 (0.597-0.661) | 0.579 (0.527-0.631) |
| <1.0 WS | 0.727 (0.697-0.757) | 0.753 (0.709-0.798) |
| ≥1 ADL | 0.627 (0.596-0.659) | 0.610 (0.559-0.661) |
| ≥1 IADL | 0.653 (0.622-0.684) | 0.666 (0.617-0.716) |

AUROC: area under the Receiver Operating Characteristic curve, CD: chronic diseases, WS: walking speed (m/s), MMSE: Mini Mental State Examination, ADL: activities of daily living, IADL: instrumental activities of daily living.

All estimates derived from logistic regressions adjusted by sex, age and education.
